# BiMPADR: A Deep Learning Framework for Predicting Adverse Drug Reactions in New Drugs

**DOI:** 10.3390/molecules29081784

**Published:** 2024-04-14

**Authors:** Shuang Li, Liuchao Zhang, Liuying Wang, Jianxin Ji, Jia He, Xiaohan Zheng, Lei Cao, Kang Li

**Affiliations:** Department of Biostatistics, School of Public Health, Harbin Medical University, Harbin 150081, China; 202101035@hrbmu.edu.cn (S.L.); zhangliu@hrbmu.edu.cn (L.Z.); wangliuying@hrbmu.edu.cn (L.W.); jasper0128@hrbmu.edu.cn (J.J.); 2019020102@hrbmu.edu.cn (J.H.); 2023020222@hrbmu.edu.cn (X.Z.)

**Keywords:** drug discovery, adverse drug reaction prediction, message passing neural network, BET inhibitor

## Abstract

Detecting the unintended adverse reactions of drugs (ADRs) is a crucial concern in pharmacological research. The experimental validation of drug–ADR associations often entails expensive and time-consuming investigations. Thus, a computational model to predict ADRs from known associations is essential for enhanced efficiency and cost-effectiveness. Here, we propose BiMPADR, a novel model that integrates drug gene expression into adverse reaction features using a message passing neural network on a bipartite graph of drugs and adverse reactions, leveraging publicly available data. By combining the computed adverse reaction features with the structural fingerprints of drugs, we predict the association between drugs and adverse reactions. Our models obtained high AUC (area under the receiver operating characteristic curve) values ranging from 0.861 to 0.907 in an external drug validation dataset under differential experiment conditions. The case study on multiple BET inhibitors also demonstrated the high accuracy of our predictions, and our model’s exploration of potential adverse reactions for HWD-870 has contributed to its research and development for market approval. In summary, our method would provide a promising tool for ADR prediction and drug safety assessment in drug discovery and development.

## 1. Introduction

Adverse drug reactions (ADRs), according to the WHO, are any harmful or unintended responses to a medication occurring at normal doses used for disease prevention, diagnosis, or treatment [1]. Adverse drug reactions (ADRs) pose a substantial challenge in contemporary drug discovery and are a major contributor of illness and mortality in healthcare [2]. ADRs have been identified as the fourth leading cause of death in the United States. Annually, statistics show that nearly 100,000 fatalities are attributed to adverse drug reactions (ADRs) resulting from the use of medications at their recommended dosages [3,4,5]. ADRs also impose a significant financial burden on public health systems. Studies have shown that the incremental total cost per patient attributed to ADRs ranges from approximately EUR 702 to EUR 7318 [6,7]. Moreover, ADRs play a prominent role in the failure of drug research. Safety-related concerns are responsible for 35% of drug failures in Phase I and 28% in Phase II, significantly impacting the progression to the drug submission stage [8,9]. The identification of ADRs for numerous drugs often occurs several years after their market introduction. Each year, the FDA withdraws drugs from the market due to adverse effects, with prominent instances including Vioxx, Fen-Phen, and Rosiglitazone [9,10]. Hence, early evaluation of potential drug adverse reactions is vital to minimize health risks for participants and to reduce drug development costs.

The conventional approach to predicting ADRs typically entails researchers engaging in pharmacological experiments or conducting clinical observations. These processes require numerous in vitro screening and in vivo preclinical animal studies. Even though these methods are time-intensive and resource-heavy, numerous ADRs of novel drugs frequently remain undiscovered [11,12]. In recent years, there has been significant progress in the development of computational prediction methods, particularly deep learning techniques, for predicting drug adverse reactions using drug-related databases.

A commonly used group of methods for predicting adverse drug reactions involve treating the problem as the inference of missing connections within a bipartite network that links drugs and side effects. Cami et al. (2011) developed a model named PPNs (predictive pharmacosafety networks), which integrates the network structure formed by known adverse drug event (ADE) relationships with specific drug information and adverse event data to predict potential unidentified ADEs [13]. Zhang et al. (2016) investigated the prediction of potential drug side effects by utilizing two recommender methods and integrating their proposed approaches with existing methods to develop ensemble models [14]. Galeano et al. (2018) proposed a recommender system that predicts drug side effects for marketed drugs using collaborative filtering algorithms [15]. Lin et al. (2013) proposed a network-based external link prediction method that utilizes the neighborhood of a drug in a bipartite network to infer potential adverse drug reactions [16].

Another group of widely adopted methods employ multisource data to predict the associations between drugs and adverse reactions. Yamanishi et al. (2012) presented a drug side effect prediction approach that integrates chemical and biological spaces based on kernel regression models [17]. Liu et al. (2012) utilized five machine learning algorithms for predicting adverse drug reactions by leveraging the chemical, biological, and phenotypic properties of drugs [18]. Zhang et al. (2015) proposed a feature selection-based multi-label k-nearest neighbor method, which adopts ensemble learning techniques to combine various drug related features [19]. Ding et al. (2018) identified drug–side effect associations using a combination of a semi-supervised model and multiple kernel learning. Their approach enabled the integration of multiple sources of drug-related information, including the known relationships between drugs and side effect terms [20].

Although previous methods have yielded promising predictive outcomes, they encounter challenges when applied to new drugs with limited pre-existing information. Specifically, the approach relying on known neighbor nodes in the constructed heterogeneous graph fails to predict the potential adverse drug reactions (ADRs) for such scenarios. Moreover, the early stages of drug development mainly offer information on the chemical structure of the drug candidate, while certain biological information cannot be incorporated into the prediction model. Consequently, these methods do not provide prediction frameworks suitable for new drug molecules.

Obviously, there are also methods developed for predicting adverse reactions of new drugs. Pauwels et al. (2011) employed a sparse canonical correlation analysis model that relied on chemical structures to predict potential drug side effects [21]. Niu et al. (2015) developed a web service called DSEP, which utilizes chemical substructures to predict potential adverse drug reactions (ADRs) without relying on other factors [22]. Dimitri et al. (2017) introduced DrugClust, a method that clusters drugs based on their features and subsequently predicts side effects using Bayesian scores [23]. Ping Xuan et al. (2022) explored the effective utilization of graph structures and attribute information in drug-related data for predicting drug side effects. By considering the relationships between drugs, drug features, and side effect labels, they proposed a novel approach to enhance the accuracy of side effect prediction [24].

However, these methods exhibit limitations, including the random allocation of drug–adverse reaction pairs into training and testing sets. This approach leads to the inadvertent use of information from test set drugs during training and a deficiency in external validation. Furthermore, these methods have not fully utilized the potential of drug gene expression profile data. Some studies indicate that drug-induced alterations in gene expression may contribute to systemic off-target effects and subsequent adverse effects [25,26,27,28]. This highlights the potential significance of transcriptomic data, where alterations in gene expression can act as early markers of toxicity. These changes are frequently detectable before the appearance of histopathological or clinical signs, offering crucial insights into drug adverse reactions [29].

To overcome the limitations of the previously mentioned methods, we propose BiMPADR, a deep learning framework designed for predicting adverse drug reactions (ADRs) in new drugs. We hypothesized that compounds with similar structures are likely to elicit analogous adverse reactions. Differential gene expression levels can lead to different adverse reactions. Our framework incorporates a binary network-based message passing neural network that integrates drug expression signatures related to each ADR into its feature representation. These features are subsequently merged with compound structural data, represented by fingerprints, and a fully connected neural network is utilized to predict the associations between drugs and ADRs. Extensive evaluations on various representative datasets confirm the high accuracy of our method. Furthermore, the performance on external validation data showcases the utility of our model as a highly valuable tool for predicting ADRs in new drugs.

## 2. Results and Discussion

### 2.1. Performance on Different Datasets

We present all the results of our model in Table 1, which includes the performances on the training set, test set, and external validation dataset. It can be observed that regardless of the fingerprint used, the model consistently demonstrates stable and satisfying predictive performances across all four data sources. In the case of the external validation dataset, the AUC exceeds 0.85. The Precision of the model in the test set can reach 0.785~0.855. In purely external validation, the Precision drops slightly because this part of the data uses extremely unbalanced data. However, the AUC considers the overall performance of the classifier at different thresholds, not just the accuracy at a single threshold. Therefore, the AUC is still relatively high when the Precision is low, indicating that the model still has a good sorting ability when distinguishing between majority and minority classes; it does not affect the effect of our model in clinical application.

**Figure 1:**
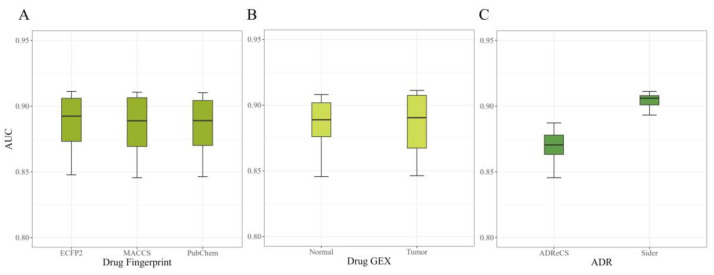
AUC of the external validation dataset under different conditions: (**A**) different compound fingerprint selections; (**B**) different drug cell line expression data selections; (**C**) different adverse reaction selections.

To further explore the factors influencing the model’s performance and its applicability range, we depict the results of the model under different input conditions (AUC on the external validation dataset) using a box plot in Figure 1. The following results can be derived from the analysis:

#### 2.1.1. Performance on Different Fingerprints

Different types of drug fingerprints may have different calculation methods and thus different representational capabilities. Based on the results shown in Figure 1A, we observed that the choice of different compound fingerprints as drug structural features during model training did not significantly impact the model’s performance. Therefore, we can conclude that the widely applied fingerprints that represent compound structural features can be effectively utilized in our model without excessive consideration of specific fingerprint selection or conversion. This finding also highlights the robustness of our model in handling diverse types of compound data.

#### 2.1.2. Performance on Different GE

Accurate prediction results can be obtained regardless of the type of cell line used for modeling, but the shorter length of the box plot from Figure 1B for normal cell lines indicates greater stability in the results. It can be inferred that certain gene perturbations after drug treatment may lead to the occurrence of adverse reactions, and these perturbations are relatively similar between normal and tumor cell lines. Therefore, in the absence of gene expression data from normal cell lines, gene perturbation data from tumor cell lines can also be widely applicable in adverse reaction prediction research.

#### 2.1.3. Performance on ADR Selection

When we selected all adverse reactions from SIDER, the AUC was above 0.9, while choosing adverse reactions that appeared in the ADReCS dataset resulted in an AUC of around 0.86 (Figure 1C). One possible reason for this result could be that there is less association between the adverse reactions provided by ADReCS and the 978 core landmark genes, with most associations being filled with zeros. Another reason could be that constructing a dataset by directly selecting all adverse reactions from SIDER provides more drug–adverse reaction pairs, a larger sample size, and a better fitting of the model. Whether the initial information related to adverse reaction genes contributes to the prediction needs to be further explored through ablation experiments.

### 2.2. Ablation Study

We conducted ablation experiments to explore the impact of the selection of initial information related to adverse reactions and the application of the MPNN module on the predictive performance of the model. Since the choice of different compound fingerprints had a minimal impact on the model, we did not consider the role of fingerprints in this part of this study.

To explore whether using ADR–gene association information as the initial input feature can improve the model’s performance, we conducted two variant studies:

1. The first variant involved replacing the initial feature vectors of adverse reactions with zero vectors, completely excluding the use of ADR–gene association information.
2. The second variant maintained the same input as the original model but only utilized this information during the computation of attention coefficients in the binary network information propagation, without incorporating the adverse reaction initial features in the information update function, denoted as $h_{v_{j}}=ReLU(m_{v_{j}})$. The difference in this process lies in the addition of a self-loop, where the original method is set to TRUE, while the ablation experiments are set to FALSE.

**Figure 2:**
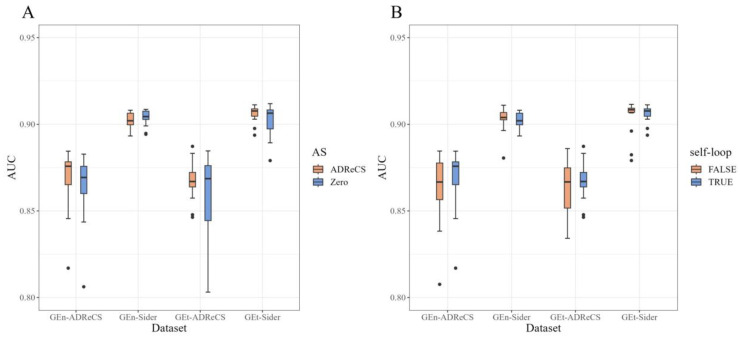
AUC of the external validation dataset under different ablations: (**A**) ablation experiments without ADR–gene information; (**B**) ablation experiments without self-loop.

Table 2 and Table 3 present the results of the two ablation experiments in the external dataset, and Figure 2 provides a comparison between our method and the results of the ablation experiments. From Figure 2A, it can be observed that replacing the original features with zero vectors did not significantly degrade the model’s performance. However, the AUC values fluctuated more, and the stability slightly decreased under different conditions. Figure 2B also demonstrates a similar trend, but when the sample size is sufficiently large, such as when training the model using the GEn-SIDER and GEt-SIDER datasets, the impact of adding self-loops is not substantial. Therefore, we can infer that the adverse reaction–gene association information obtained from the ADReCS database can improve the predictive accuracy and stability of the model to some extent. However, when a particular adverse reaction does not exist in that database and we still want to understand its likelihood of occurrence, we can use a zero feature vector as its input in the model.

**Figure 3:**
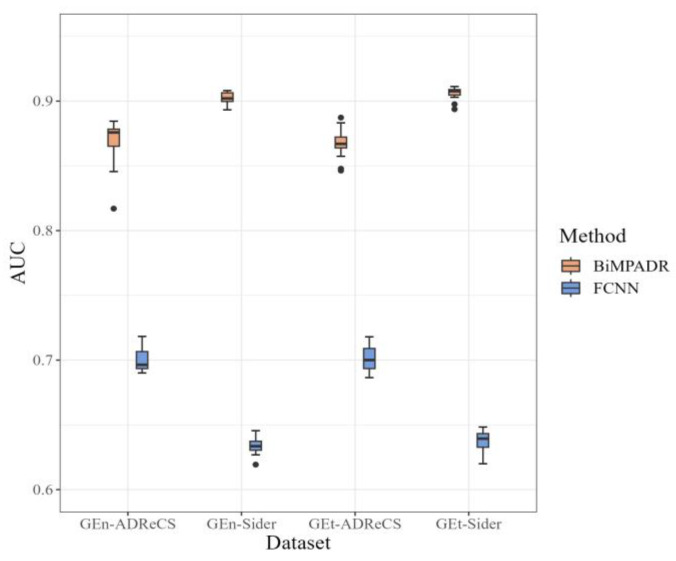
AUC of the external dataset under ablation experiments without MPNN module.

In order to investigate whether the MPNN module effectively utilizes the gene expression information of drugs and its impact on model performance, we directly concatenated the compound structure features with the adverse reaction–gene association features and used a fully connected neural network (FCNN) for prediction. From Table 4 and Figure 3, it can be observed that the predictive performance of the model significantly decreases without utilizing the MPNN module to integrate the gene expression information of drugs into the adverse reaction features. Additionally, compared to the original method, using a dataset constructed with all adverse reactions from the SIDER database, although having a larger sample size, yields poorer prediction results. This experiment demonstrates the crucial role of drug-induced cell line gene expression information in predicting associations between drugs and adverse reactions. Furthermore, the information integration method used in our model effectively utilizes the relevant information.

### 2.3. Performance of BiMPADR Compared with State-of-the-Art Methods

To ensure comparability between models, we select existing methods that can predict adverse reactions based solely on compound structure, including Pauwels’s method (SCCA) [21] and DrugClust [23]. These two comparison methods and the predictive performance of our model are shown in Table 5.

**Figure 4:**
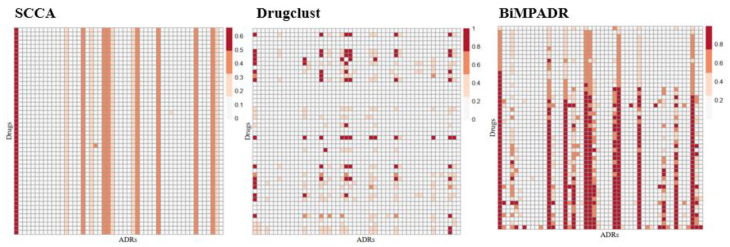
Visualization of predicted values on GEn-SIDER datasets by three methods.

By comprehensive comparison, the AUC value of the SCCA algorithm is above 0.89, slightly higher than that of the BiMPADR algorithm, 0.86, but its ACC value is only about 0.5, which is far lower than the predicted result of this model. The accuracy of the model is also low, with a minimum of 0.38. The AUC value of the DrugClust algorithm is about 0.6, which is much lower than the other two methods. Although its Precision is relatively high, we tend to pay more attention to the AUC index, which can reflect the ordering ability in clinical practice. We randomly selected 50 drugs and 50 adverse reactions from the predicted values of each method in GEn-SIDER datasets to draw heat maps, and the results are shown in Figure 4. As can be seen from the graph, the SCCA and DrugClust prediction results have multiple lines of identical data. This reflects a very big drawback of the two control models; that is, multiple drugs often have the same predictive value vector, and the prediction results of multiple drugs for each adverse reaction may be the same, which greatly reduces the practicality of the prediction model in clinical research.

### 2.4. Case Study

**Figure 5:**
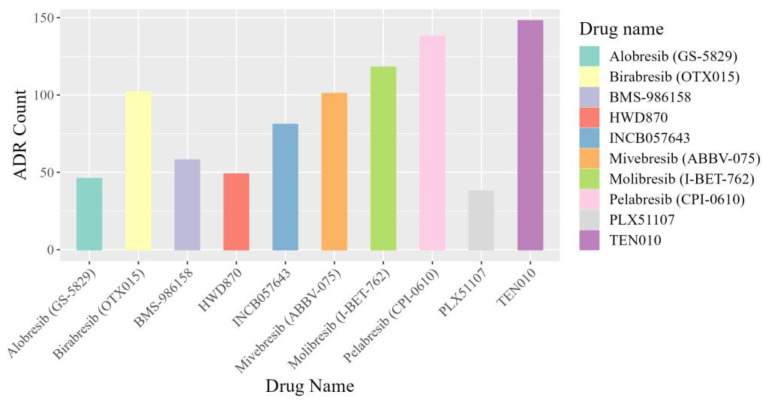
Count of ADRs with a predicted value greater than 0.99.

We performed a case study to evaluate the accuracy of our model’s novel predictions by conducting a literature-based assessment of the newly identified associations. NHWD-870 [30] is a novel and potent BET inhibitor intended for the treatment of various solid tumors. We used the best performance model to predict the adverse reactions of NHWD-870 and nine other BET inhibitors, Alobresib [31], INCB0576543 [32], Mivebresib [33], Pelabresib [34], Birabresib [35], Molibresib [36], TEN010 [37], PLX51107 [38], and BMS-986158 [39], that have undergone Phase I/II clinical trials. The selected drugs were not present in our modeling dataset. The complete prediction results can be found in the Supplementary Section S1. Figure 5 shows the number of adverse drug reactions with predicted values higher than 0.99. From the graph, it can be observed that HWD-870 is associated with fewer adverse reactions, and it has fewer reactions than BMS-986158.

We present the top ten adverse reactions for each drug and validate the accuracy of our predictions through the public verification of clinical trial research results on NIH (https://ncbi.nlm.nih.gov/, accessed on 12 December 2023.). Additionally, the adverse reactions on the blood and lymphatic systems recorded in the NIH are important factors that affect the development and application of BET inhibitors. Therefore, we discuss the predicted values obtained through our model for the blood and lymphatic systems-related adverse reactions documented in the NIH. The results of BMS-986158 [39] are shown below, which are most similar to NHWD-870. Other detailed results evidenced by the NIH can be found in Supplementary Section S2.

From Table 6, it can be observed that for BMS-986158, almost all predicted top ten adverse reactions were found in the corresponding clinical reports’ adverse events. BMS-986158 may potentially lead to rhabdomyolysis, although no supporting literature has been found. Regarding BMS potentially causing hyperlipidemia, there is relevant research suggesting that the BET inhibitor Apabetalone can lead to an increase in HDL-C, which contradicts our predicted results. Therefore, we used our model to calculate the association score between Apabetalone and hyperlipidemia, which resulted in a score of 0.46. Consequently, BMS may have a higher cardiovascular risk compared to other BET inhibitors. From Table 7, adverse reactions related to the blood and lymphatic systems also had predicted values mostly exceeding 0.5, even reaching above 0.9.

**Figure 6:**
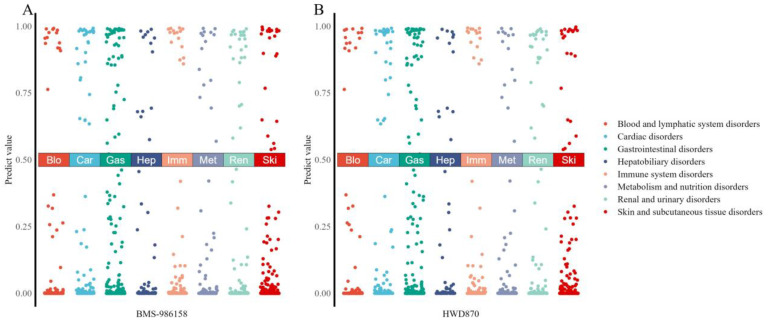
Adverse reaction predictions across different organ system classifications: (**A**) predictive value for BMS-986158 in different system; (**B**) predictive value for HWD-870 in different system.

Since NHWD-870 is a structural modification of BMS, we provide an overview of the adverse reactions produced by these two drugs in different organ systems, as shown in Figure 6 (results of other drugs can be found in Supplementary Section S2). The more clustered the points are at the top, the more likely the drug is to generate a greater number of adverse reactions within that system. It can be observed that NHWD-870 exhibits reduced adverse reactions in the blood and lymphatic system compared to BMS. However, it may potentially cause more adverse reactions in the liver and renal system.

**Figure 7:**
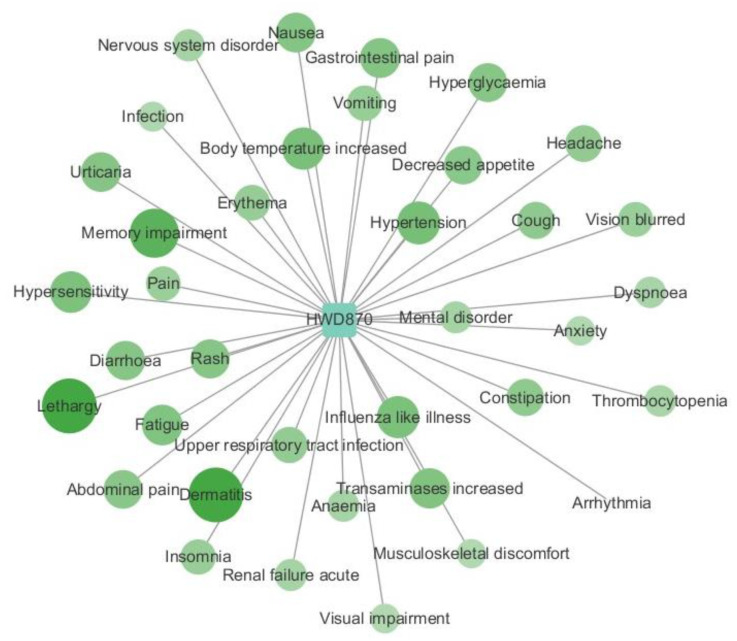
Most relative ADRs of NHWD-870.

For HWD-870, we selected adverse reactions with predicted values > 0.99 and created an association network shown in Figure 7 using the software ‘Cytoscape 3.6.1’. According to our predictions, HWD-870 is associated with common blood and lymphatic system disorders, such as Anemia, Thrombocytopenia, Coagulopathy, Neutropenia, and Leukopenia. It may also cause other severe adverse reactions in different systems, such as Acute Renal Failure, Upper Respiratory Tract Infection, and Hypertension.

## 3. Materials and Methods

### 3.1. Datasets

In this study, we use four types of data sources: (1) ground truth for drug–ADR pair labels, (2) gene expression profiling of the compounds (GE), (3) the chemical structure of the compounds (CS), and (4) ADR–gene associations (AS).

We obtained the ADR labels from the SIDER 4.1 Database [40], which includes data on medications available in the market and their reported ADRs obtained from public documents. In the SIDER 4.1 version of the database, there are approximately 1430 drugs, 5868 ADRs, and 139,756 drug–ADR associations. The MedDRA concept type was used to specify ADR terms and phrases. The preferred term (PT) level in SIDER was utilized as the standard ADR vocabulary to avoid the semantic redundancy.

The Library of Integrated Network-based Cellular Signatures (LINCS) database has a large collection of gene expression profiles that show how different human cell lines respond to 20,413 compounds at the transcriptomic level [41,42]. Considering that adverse reactions often occur within the normal organs of the human body, we categorized the expression data of drugs into perturbations in normal/primary cell lines and tumor cell lines, named GEn and GEt in our research. To avoid information redundancy, we selected the strongest signatures for each drug, irrespective of the cell type, dosage, or time point, utilizing level 5 data. The signatures for the 978 directly measured landmark genes were selected in this study.

The 2D chemical structures of small-molecule compounds are represented in the SMILES format. SMILES strings for marketed drugs were collected from PubChem [43] using PubChem Compound IDs from SIDER. Drug chemical structures were mapped to three types of fingerprints: PubChem, MACCS, and ECFP using the PyBioMed [44] Python library. PubChem fingerprints consist of 881 chemical substructures derived from the PubChem database. MACCS fingerprints consist of 166 structural keys representing molecular features. ECFP fingerprints capture local and global molecular features through atom neighborhood enumeration and hashing. The fingerprint size used here is 1024 bits. 

The ADReCS-Target [45] database offers extensive information regarding ADRs resulting from drug interactions with proteins, genes, genetic variations, and gene–ADR associations. There are 1156 ADRs, 8571 genes, and 2,443,256 gene–ADR pairs included. We organized the associations between ADRs and the 978 landmark genes mentioned in the LINCS database into a binary profile. If an ADR–gene association was documented in the ADReCS-Target database, we marked that position as 1; otherwise, it was filled with 0. 

**Figure 8:**
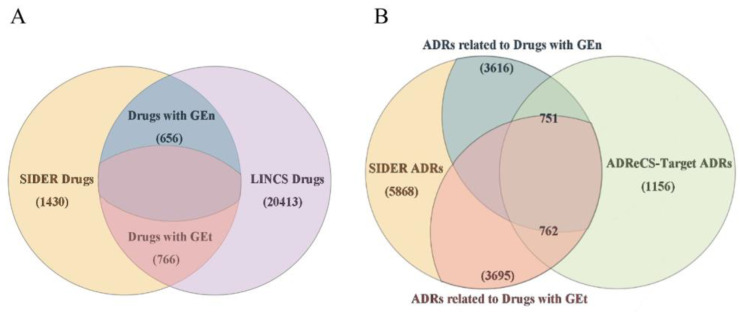
Overview of the datasets used in this study: (**A**) the drugs selected for this study; (**B**) the adverse reactions selected for this study.

The set of drugs have perturbations in the above two categories of cell lines, which can be found in SIDER, which contains 656 and 766 compounds, respectively (duplicates are avoided by taking the drug ids, which are unique). Drugs lacking gene expression information in SIDER were considered as external validation data. The ADRs that are observed with at least one drug are included. Therefore, the number of adverse reactions left for further study corresponding to these two sets of drugs is 3616 and 3695, respectively. Among these adverse reactions, 751 and 762 are also recorded in the ADReCS-Target database. In the end, we obtained a total of four datasets with varying numbers of drugs and adverse reactions (Figure 8 and Table 8).

### 3.2. Methods

#### 3.2.1. MPNNs

Message passing neural networks [46] (MPNNs) are a class of general frameworks used for supervised learning on graphs. They are commonly applied to undirected graphs, where node features are represented as $x_{v}$ and edge features as $e_{vw}$. The usage of such models primarily consists of two stages: the message passing stage and the readout stage. During the message passing stage, the model iteratively updates the hidden layer features of each node, using an information function $M_{t}$ and a vertex update function $U_{t}$, for a total of T iterations. The updated hidden layer features $h_{v}^{t}$ for each node, based on the information $m_{v}^{t+1}$ and the previous hidden layer features, can be expressed by the following formula:

(1) $$m_{v}^{t+1}=\sum_{w\in N(v)}M_{t}(h_{v}^{t},h_{w}^{t},e_{vw})$$

(2) $$h_{v}^{t+1}=U_{t}(h_{v}^{t},m_{v}^{t+1})$$

In the summation process, $N_{\left(v\right)}$ represents all neighboring nodes of the node v in the graph. During the readout stage, a common readout function R is used to calculate a feature vector based on the entire graph, according to the following formula:

(3) $$\hat{y}=R(\{h_{v}^{T}|v\in G\})$$

The message functions $M_{t}$, vertex update functions $U_{t}$, and readout function R are all learned differentiable functions. We can define these functions according to our purposes.

#### 3.2.2. Overall Schema of the Deep Learning Network

**Figure 9:**
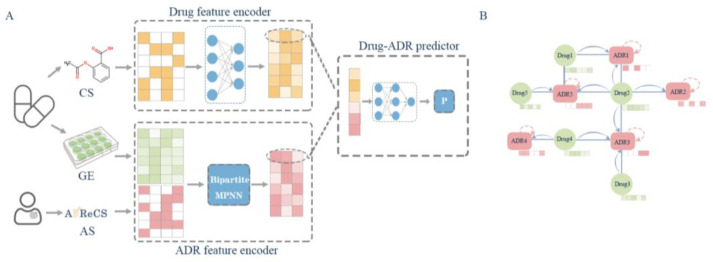
The workflow and architecture of BiMPADR: (**A**) the model receives three parts of data, chemical structures (CSs) used to encode the feature of drugs, drug-induced gene expression (GE), and ADR–gene associations (ASs) used to encode the feature of ADRs through MPNN module; (**B**) message transfer direction in the MPNN module. Solid arrows represent the transmission of drug information to adjacent adverse reactions, while dashed arrows represent the self-transmission of adverse reaction information.

In our study, we defined the task of predicting the association between drugs and adverse drug reactions (ADRs) as a binary classification problem. We extracted informative features from both drugs and ADRs and utilized these features to train the model in order to predict novel associations. Figure 9 shows the frame of our method. We generated the features of ADRs via MPNNs and yielded a latent representation of drug fingerprints via fully connected layers. After processing both the drug and ADR layers, we concatenated these layers and constructed the fully connected layer, resulting in the output. Every layer except the output layer was activated with the LeakyReLU function. The output layer was activated with the sigmoid function to predict whether the drug and ADR interact.

#### 3.2.3. MPNN Layer with ADR Embedding Vector

We can view the association network between drugs and adverse reactions as a bipartite graph BG(U,V,E), where U represents the drug nodes in the graph; V represents the adverse reaction nodes; $u_{i}$ and $v_{j}$ denote the i-th and j-th node in U and V, respectively; i=1,2,…,M, j=1,2,…,N; E is a set of edges representing an association between a drug and an adverse drug reaction; e={(u,v)|u∈U, v∈V}; and $e_{ij}$ denotes the edge between $u_{i}$ and $v_{j}$. The gene expression feature matrix for drugs can be represented as $X_{u}$, $X_{u}\in R^{M\times P}$, where $\vec{x_{u_{i}}}$ represents the gene expression feature vectors for each drug. The initial input feature matrix for adverse reactions can be represented as $X_{v}$, $X_{v}\in R^{N\times Q}$, where $\vec{x_{v_{j}}}$ represents the initial feature vectors for each adverse reaction and $h_{v_{j}}$ represents the updated adverse reaction feature vectors after information propagation.

To apply the MPNN framework on the bipartite graph, appropriate information functions and vertex update functions need to be selected for feature propagation and aggregation among the nodes. For simplicity, we perform only one iteration, denoted as T=1. The process of propagating the gene expression information from drug nodes to adverse reaction nodes’ feature representations can be defined as

(4) $$m_{v_{j}}=\sum_{u_{i}\in N_{v_{j}}^{e}}M(\vec{x_{v_{j}}},\vec{x_{u_{i}}})$$

(5) $$h_{v_{j}}=U(\vec{x_{v_{j}}},m_{v_{j}})$$

where $N_{v_{j}}^{e}$ represents all nodes connected to node $v_{j}$ through edges in the bipartite graph BG(U,V,E). We apply the GAT (Graph Attention Network) [47] to the process of information propagation and aggregation, defining $W^{u}\in R^{P\times S}$ and $W^{v}\in R^{Q\times S}$ as two learnable weight parameter matrices. The purpose is to linearly transform the input features of the two types, aiming to acquire sufficient data representation capacity. Thus, our message functions M and vertex update functions U can be expressed as

(6) $$m_{v_{j}}=\sum M(\vec{x_{v_{j}}},\vec{x_{u_{i}}})=\sum \alpha_{u_{i},v_{j}}W^{v}\vec{x_{v_{j}}}$$

(7) $$h_{v_{j}}=U\left(\vec{x_{v_{j}}},m_{v_{j}}\right)=W^{v}\vec{x_{v_{j}}}+ReLU(m_{v_{j}})$$

where $\alpha_{u_{i},v_{j}}$ represents the attention coefficients, indicating the importance of a node to node $v_{j}$. It can be calculated using the following formula, where σ is the non-linear function LeakyReLU and $\vec{\alpha }\in R^{2S}$:

(8) $$\alpha_{u_{i},v_{j}}=\frac{exp(\rho ({\vec{\alpha }}^{T}[W^{u}\vec{x_{u_{i}}}||W^{v}\vec{x_{v_{j}}}]))}{\sum_{u_{k}\in N_{v_{j}}^{e}}\rho ({\vec{\alpha }}^{T}[W^{u}\vec{x_{u_{k}}}||W^{v}\vec{x_{v_{j}}}])}$$

### 3.3. Experimental Setting

We employ 5-fold cross-validation to assess the performance of our models. The cross-validation folds are stratified based on drugs, ensuring that all experiments involving a particular drug are either entirely in the training set or completely in the test set. This setup enables our models to predict the side effects of previously unseen drugs during testing. To tackle data imbalance in the training datasets and test datasets, we consider all confirmed drug–adverse reaction associations as positive samples, and we randomly select unobserved associations as negative samples in a 1:1 ratio. In external validation datasets, we predict all possible associations between drugs and adverse events.

We utilize the binary cross-entropy [48] (BCE) loss function to measure the discrepancy between predicted and true labels. An Adam optimizer [49] is used for training the neural networks. Additionally, we incorporate regular dropout to the hidden layer units in the MLP decoder, which helps to prevent overfitting and encourages the model to learn more robust and generalizable representations.

We measure the prediction performance using three criteria: the AUC, Precision, and ACC, which are widely used for drug indication prediction tasks. Let P and N represent the counts of positive and negative instances in the dataset, respectively. TP, FN, TN, and FP denote the counts of true positives, false negatives, true negatives, and false positives in the predictions. The following performance metrics are defined:

(9) $$Precision=\frac{TP}{TP+FP}$$

(10) $$ACC=\frac{TP+TN}{P+N}$$

Our method is implemented in Python 3.7.13 and PyTorch 1.7.1. We use a Random Search to determine the hyperparameters. The batch size is set to be 10,000, and the Adam optimizer is used with a learning rate of 1 × 10^−4^. We set the dropout rate for this work to 0.2. We allow the model to run for 300 epochs at most for all datasets. The best-performing model is selected at the epoch giving the best AUC score on the test set, which is then used to evaluate the final performance on the external validation set.

## 4. Conclusions

We developed a novel ADR detection model named BiMPADR based on a bipartite message passing neural network. Our model achieved information fusion across drug gene expressions and ADR–gene association information. The proposed model conducted the integration of drug expression information and gene–ADR association information, enriching the practical significance provided by each adverse reaction feature vector. Extensive experiments have shown that our model achieves an excellent performance in the task of drug–ADR prediction under different conditions. Furthermore, we conducted external validation to confirm the potential applicability of our approach to new drugs. Case studies provide concrete examples that validate the practical utility of our approach. It will assist pharmacists and healthcare providers in comprehending the potential risks of drug side effects and addressing the problem of underreporting spontaneous reports. In future work, we intend to employ geometric deep learning techniques to extract compound structural features and better utilize compound information to further enhance the predictive performance of our model. Additionally, we aim to identify suitable methods for assessing the contribution of genes to the occurrence of each adverse reaction.

## Figures and Tables

**Table 1:** The summary of model performance.

Dataset	CS	Train			Test			External Validation		
		AUC	Precision	ACC	AUC	Precision	ACC	AUC	Precision	ACC
GEn-ADReCS	ECFP2	0.948 ± 0.015	0.839 ± 0.032	0.877 ± 0.017	0.873 ± 0.018	0.796 ± 0.034	0.802 ± 0.015	0.861 ± 0.026	0.177 ± 0.028	0.77 ± 0.053
	MACCS	0.958 ± 0.007	0.844 ± 0.016	0.889 ± 0.009	0.879 ± 0.019	0.798 ± 0.028	0.808 ± 0.015	0.871 ± 0.016	0.178 ± 0.017	0.774 ± 0.033
	PubChem	0.97 ± 0.008	0.869 ± 0.017	0.907 ± 0.013	0.894 ± 0.01	0.815 ± 0.019	0.819 ± 0.007	0.874 ± 0.007	0.193 ± 0.012	0.802 ± 0.019
GEn-SIDER	ECFP2	0.975 ± 0.012	0.89 ± 0.027	0.923 ± 0.025	0.898 ± 0.009	0.853 ± 0.011	0.831 ± 0.012	0.903 ± 0.003	0.109 ± 0.007	0.849 ± 0.013
	MACCS	0.983 ± 0.01	0.898 ± 0.028	0.937 ± 0.021	0.906 ± 0.006	0.852 ± 0.017	0.84 ± 0.003	0.903 ± 0.007	0.106 ± 0.013	0.842 ± 0.024
	PubChem	0.98 ± 0.011	0.892 ± 0.034	0.928 ± 0.027	0.909 ± 0.013	0.847 ± 0.003	0.84 ± 0.015	0.902 ± 0.003	0.105 ± 0.005	0.844 ± 0.01
GEt-ADReCS	ECFP2	0.95 ± 0.024	0.852 ± 0.03	0.882 ± 0.032	0.878 ± 0.019	0.807 ± 0.027	0.803 ± 0.023	0.872 ± 0.015	0.188 ± 0.015	0.805 ± 0.015
	MACCS	0.96 ± 0.014	0.842 ± 0.032	0.888 ± 0.022	0.877 ± 0.012	0.788 ± 0.029	0.798 ± 0.017	0.868 ± 0.01	0.168 ± 0.02	0.768 ± 0.042
	PubChem	0.966 ± 0.011	0.873 ± 0.029	0.908 ± 0.018	0.877 ± 0.013	0.813 ± 0.019	0.801 ± 0.019	0.863 ± 0.01	0.189 ± 0.019	0.808 ± 0.029
GEt-SIDER	ECFP2	0.982 ± 0.007	0.897 ± 0.024	0.934 ± 0.017	0.913 ± 0.008	0.849 ± 0.02	0.842 ± 0.009	0.907 ± 0.005	0.107 ± 0.013	0.85 ± 0.023
	MACCS	0.989 ± 0.005	0.917 ± 0.014	0.951 ± 0.01	0.91 ± 0.006	0.86 ± 0.01	0.842 ± 0.008	0.905 ± 0.007	0.11 ± 0.006	0.859 ± 0.012
	PubChem	0.99 ± 0.005	0.918 ± 0.016	0.951 ± 0.012	0.91 ± 0.005	0.865 ± 0.013	0.837 ± 0.011	0.907 ± 0.002	0.114 ± 0.008	0.864 ± 0.013

**Table 2:** Ablation experiments for BiMPADR models without ADR–gene information.

Dataset	Train			Test			External Validation		
	AUC	Precision	ACC	AUC	Precision	ACC	AUC	Precision	ACC
GEn-ADReCS	0.953 ± 0.02	0.851 ± 0.033	0.887 ± 0.03	0.878 ± 0.018	0.804 ± 0.02	0.808 ± 0.017	0.864 ± 0.019	0.184 ± 0.018	0.789 ± 0.025
GEn-SIDER	0.984 ± 0.012	0.906 ± 0.034	0.939 ± 0.026	0.904 ± 0.009	0.855 ± 0.016	0.836 ± 0.006	0.904 ± 0.005	0.109 ± 0.01	0.849 ± 0.018
GEt-ADReCS	0.937 ± 0.026	0.823 ± 0.043	0.864 ± 0.032	0.871 ± 0.022	0.785 ± 0.035	0.8 ± 0.018	0.858 ± 0.026	0.167 ± 0.027	0.765 ± 0.052
GEt-SIDER	0.98 ± 0.017	0.897 ± 0.023	0.933 ± 0.026	0.911 ± 0.012	0.849 ± 0.012	0.843 ± 0.011	0.902 ± 0.01	0.103 ± 0.008	0.845 ± 0.016

**Table 3:** Ablation experiments for BiMPADR models without self-loop.

Dataset	Train			Test			External Validation		
	AUC	Precision	ACC	AUC	Precision	ACC	AUC	Precision	ACC
GEn-SIDER	0.978 ± 0.016	0.892 ± 0.028	0.927 ± 0.027	0.906 ± 0.009	0.852 ± 0.015	0.839 ± 0.007	0.903 ± 0.007	0.108 ± 0.01	0.848 ± 0.018
GEn-ADReCS	0.953 ± 0.027	0.851 ± 0.04	0.888 ± 0.037	0.875 ± 0.019	0.801 ± 0.023	0.805 ± 0.016	0.863 ± 0.02	0.182 ± 0.019	0.785 ± 0.03
GEt-SIDER	0.982 ± 0.012	0.903 ± 0.033	0.937 ± 0.022	0.914 ± 0.01	0.856 ± 0.025	0.844 ± 0.007	0.904 ± 0.01	0.11 ± 0.018	0.854 ± 0.029
GEt-ADReCS	0.951 ± 0.018	0.847 ± 0.031	0.886 ± 0.026	0.878 ± 0.014	0.803 ± 0.02	0.81 ± 0.012	0.864 ± 0.015	0.179 ± 0.017	0.788 ± 0.026

**Table 4:** Ablation experiments for BiMPADR models without MPNN module.

Dataset	Train			Test			External Validation		
	AUC	Precision	ACC	AUC	Precision	ACC	AUC	Precision	ACC
GEn-SIDER	0.802 ± 0.011	0.719 ± 0.009	0.716 ± 0.008	0.649 ± 0.023	0.659 ± 0.03	0.608 ± 0.02	0.634 ± 0.007	0.038 ± 0.003	0.755 ± 0.032
GEn-ADReCS	0.877 ± 0.016	0.753 ± 0.024	0.775 ± 0.015	0.716 ± 0.01	0.667 ± 0.014	0.643 ± 0.01	0.7 ± 0.009	0.103 ± 0.005	0.712 ± 0.033
GEt-SIDER	0.798 ± 0.011	0.718 ± 0.012	0.713 ± 0.008	0.651 ± 0.019	0.67 ± 0.034	0.606 ± 0.016	0.638 ± 0.008	0.039 ± 0.003	0.771 ± 0.041
GEt-ADReCS	0.879 ± 0.019	0.755 ± 0.018	0.777 ± 0.015	0.717 ± 0.012	0.67 ± 0.017	0.642 ± 0.01	0.701 ± 0.01	0.1 ± 0.006	0.712 ± 0.037

**Table 5:** Performance comparison of different approaches.

Dataset	Method	AUC	Precision	ACC
GEn-SIDER	DrugClust	0.6044 ± 0.0111	0.1877 ± 0.0177	0.9644 ± 0.003
	SCCA	0.9131 ± 0.0002	0.0392 ± 0.0008	0.4814 ± 0.0121
	BiMPADR	0.902 ± 0.003	0.105 ± 0.005	0.844 ± 0.01
GEn-Adrecs	DrugClust	0.615 ± 0.0169	0.2415 ± 0.0243	0.913 ± 0.0086
	SCCA	0.8891 ± 0.0005	0.1091 ± 0.0014	0.5468 ± 0.0066
	BiMPADR	0.874 ± 0.007	0.193 ± 0.012	0.802 ± 0.019
GEt-SIDER	DrugClust	0.6335 ± 0.0169	0.2087 ± 0.0283	0.9662 ± 0.0017
	SCCA	0.9137 ± 0.0005	0.0381 ± 0.0009	0.4736 ± 0.0128
	BiMPADR	0.907 ± 0.002	0.114 ± 0.008	0.864 ± 0.013
GEt-Adrecs	DrugClust	0.651 ± 0.0202	0.2498 ± 0.0195	0.9125 ± 0.0042
	SCCA	0.8897 ± 0.0004	0.1061 ± 0.0005	0.5485 ± 0.0022
	BiMPADR	0.863 ± 0.01	0.189 ± 0.019	0.808 ± 0.029

**Table 6:** Evidence for the top ten predicted ADRs in example drugs.

Drug Name	ADR Name	Pred Value	NCT Number
BMS-986158	Transaminases increased	0.998	NCT02419417
	Rhabdomyolysis	0.998	
	Dermatitis	0.997	NCT02419417
	Intermittent claudication	0.997	NCT02419417
	Hypertriglyceridaemia	0.997	
	Hyperglycaemia	0.996	NCT02419417
	Hyperlipidaemia	0.996	
	Upper respiratory tract infection	0.996	NCT02419417
	Influenza-like illness	0.996	NCT02419417
	Gastroenteritis	0.995	NCT02419417

**Table 7:** Blood and lymphatic system disorders ADRs recorded by NIH.

Drug Name	ADR Name	Pred Value	NCT Number
BMS-986158	Anemia	0.991	NCT02419417
	Leukopenia	0.983	NCT02419417
	Lymphopenia	0.689	NCT02419417
	Neutropenia	0.985	NCT02419417
	Thrombocytopenia	0.991	NCT02419417

**Table 8:** Summary of datasets used in this study.

Dataset	Number of Drugs	Number of ADRs	Number of Drugs in External Dataset
GEn-SIDER	656	3616	774
GEn-ADReCS	656	751	774
GEt-SIDER	766	3695	664
GEt-ADReCS	766	762	664

## Data Availability

The datasets and codes used during the current study are available in the github repository, https://github.com/Ls94wood/BiMPADR.git (accessed on 9 December 2021).

## Supplementary Materials

The following supporting information can be downloaded at https://www.mdpi.com/article/10.3390/molecules29081784/s1, Predicted values for all ten BET inhibitors across 3616 adverse drug reactions. Table S1: Evidence for the top ten predicted ADRs. Table S2: Blood and lymphatic system disorder ADRs recorded by NIH and related literature. Figure S1: Adverse reactions produced by other BET inhibitors in different organ systems. Refs. [50,51,52,53] are cited in supplementary files.
