# Supplementary material for: BiMPADR: A Deep Learning Framework for Predicting Adverse Drug Reactions in New Drugs
Source: Molecules. 2024 Apr 14;29(8):1784. doi: 10.3390/molecules29081784 (PMC11051887; doi:10.3390/molecules29081784)
Supplement: Supplementary file 1 [file molecules-29-01784-s001.zip › molecules-2952776-supplementary/Supplementary Section2.pdf]

Table S1. Evidence for the top ten predicted ADRs.

Drug name	ADR name	Pred value	NCT Number
**Birabresib (OTX015)**	Dermatitis	0.9989	NCT01713582, NCT02296476, NCT02259114
	Body temperature increased	0.9986	NCT01713582, NCT02259114
	Hypertension	0.9985	NCT01713582, NCT02296476, NCT02259114
	Thrombocytopenia	0.9982	NCT01713582, NCT02296476, NCT02259114
	Decreased appetite	0.9978	NCT01713582, NCT02259114
	Musculoskeletal discomfort	0.9978	NCT01713582, NCT02296476, NCT02259114
	Anaemia	0.9977	NCT01713582, NCT02259114
	Infection	0.9977	NCT01713582, NCT02296476, NCT02259114
	Arthralgia	0.9975	NCT01713582, NCT02259114
	Insomnia	0.9974	NCT01713582, NCT02296476, NCT02259114
**INCB057643**	Dermatitis	0.9991	NCT02959437
	Hypersensitivity	0.9984	
	Urticaria	0.9984	NCT02959437, NCT02711137
	Body temperature increased	0.9978	NCT02959437, NCT02711137
	Asthenia	0.9976	NCT02959437, NCT02711137
	Hypertension	0.9976	NCT02711137
	Decreased appetite	0.9975	NCT02959437, NCT02711137
	Pain	0.9973	NCT02959437, NCT02711137
	Fatigue	0.9972	NCT02959437, NCT02711137
	Insomnia	0.9970	NCT02959437, NCT02711137
**GSK525762A Molibresib (I-BET-762)**	Hypertension	0.9987	NCT01943851, NCT01587703
	Oedema peripheral	0.9983	NCT01943851, NCT01587703
	Body temperature increased	0.9981	
	Alopecia	0.9981	NCT01587703
	Dermatitis	0.9981	NCT01943851, NCT01587703
	Haemorrhage	0.9980	NCT01943851, NCT01587703
	Pneumonia	0.9979	NCT01943851, NCT01587703
	Neuropathy peripheral	0.9979	NCT01587703
	Cough	0.9979	NCT01943851, NCT01587703
	Anxiety	0.9979	NCT01943851, NCT01587703
**Alobresib (GS-5829)**	Dermatitis	0.9975	NCT02983604
	Fatigue	0.9943	NCT02607229, NCT02983604, NCT02392611
	Hypertension	0.9943	NCT02607229
	Hypersensitivity	0.9942	
	Asthenia	0.9939	NCT02607229, NCT02983604
	Gastrointestinal pain	0.9935	NCT02607229
	Influenza like illness	0.9935	NCT02607229, NCT02983604
	Abdominal pain	0.9934	NCT02607229, NCT02983604, NCT02392611
	Visual impairment	0.9931	NCT02392611
	Cough	0.9930	NCT02607229, NCT02983604, NCT02392611

Table S2. Blood and lymphatic system disorders ADRs recorded by NIH and related literature.

Drug name	ADR name	Pred value	NCT Number
**Birabresib (OTX015)**	Anaemia	0.9977	NCT01713582, NCT02259114
	Disseminated intravascular coagulation	0.8282	NCT01713582 NCT02259114
	Leukocytosis	0.9627	NCT01713582
	Neutropenia	0.9962	NCT01713582
	Thrombocytopenia	0.9981	NCT01713582, NCT02296476, NCT02259114
	Febrile bone marrow aplasia, Haemorrhagic disorder	<0.5	NCT01713582 (3/138, 1/138)*
**INCB057643**	Anaemia	0.9960	NCT02959437, NCT02711137
	Leukocytosis	0.8558	NCT02959437
	Thrombocytopenia	0.9967	NCT02711137
	Leukopenia	0.9935	NCT02711137
	Lymphopenia	0.5621	NCT02711137
	Neutropenia	0.9903	NCT02711137
	Iron deficiency anaemia, Lymph node pain, Splenic infarction, Splenomegaly	<0.5	NCT02711137 (1/137, 2/137, 1/137, 1/137, 1/137)*
**TEN010**	Thrombocytopenia	0.9988	A dose escalation study of RO6870810/TEN-10 in patients with acute myeloid leukemia and myelodysplastic syndrome [1]
**GSK525762A Molibresib (I-BET-762)**	Thrombocytopenia	0.9977	NCT01943851, NCT01587703
	Febrile neutropenia	0.0261	NCT01943851 (12/111)*, NCT01587703
	Neutropenia	0.9962	NCT01943851, NCT01587703
	Leukocytosis	0.9725	NCT01943851, NCT01587703
	Anaemia	0.9975	NCT01943851, NCT01587703
	Thrombotic microangiopathy	0.0013	NCT01587703(1/196)*
	Histiocytosis haematophagic	0.0004	NCT01587703(1/196)*
**PLX51107**	Anaemia	0.9923	Phase 1 Results of Bromodomain and Extraterminal Inhibitor PLX51107 in Combination with Azacitidine in Patients with Relapsed Myeloid Malignancies [2]
	Thrombocytopenia	0.9902	
	Neutropenia	0.9888	
**Pelabresib (CPI-0610)**	Anaemia	0.9983	A Phase I Study of Pelabresib (CPI-0610), a Small-Molecule Inhibitor of BET Proteins, in Patients with Relapsed or Refractory Lymphoma [3]
	Thrombocytopenia	0.9983	
	Lymphopenia	0.9277	
**Mivebresib (ABBV-075)**	Thrombocytopenia	0.9971	First-in-human Study of Mivebresib (ABBV-075), an Oral Pan-inhibitor of Bromodomain and Extra Terminal Proteins, in Patients with Relapsed/ Refractory Solid Tumors [4]
	Anaemia	0.9970	

* This is in parentheses (Number of people with this adverse event/total number of participants in the trial), some ADRs are predicted unrelated to the drug. This could suggest the occurrence of chance events or limitations in our model when dealing with infrequent adverse reactions.

Table S3. Network architecture Settings for the BiMPADR model

Module	Layer	Type	Decscription	Input Nodes	Output Nodes
Drug feature encoder	df1	Sequential	FC[1]-BN[2]- LReLU[3]-DP[4]	881	489
	df2	Sequential	FC-BN- LReLU- DP	489	978
ADR feature encoder	af	GATConv	GATConv	(978, 978)	978
Drug-ADR Prediction	p1	Sequential	FC-BN- LReLU- DP	1956	1000
	p2	Sequential	FC-BN- LReLU- DP	1000	500
	p3	Sequential	FC-BN- LReLU- DP	500	250
	p4	Sequential	FC-Sigmoid	250	1

[1]FC(Fully Connected): connects neurons fully across layers for data transformation; [2]BN(Batch Norm): stabilizes training by normalizing layer outputs; [3]LReLU(Leaky ReLU): addresses inactive neurons, allowing a small gradient when the unit is not active; [4]DP(Drop Rate): reduces overfitting by deactivating random neurons during training.

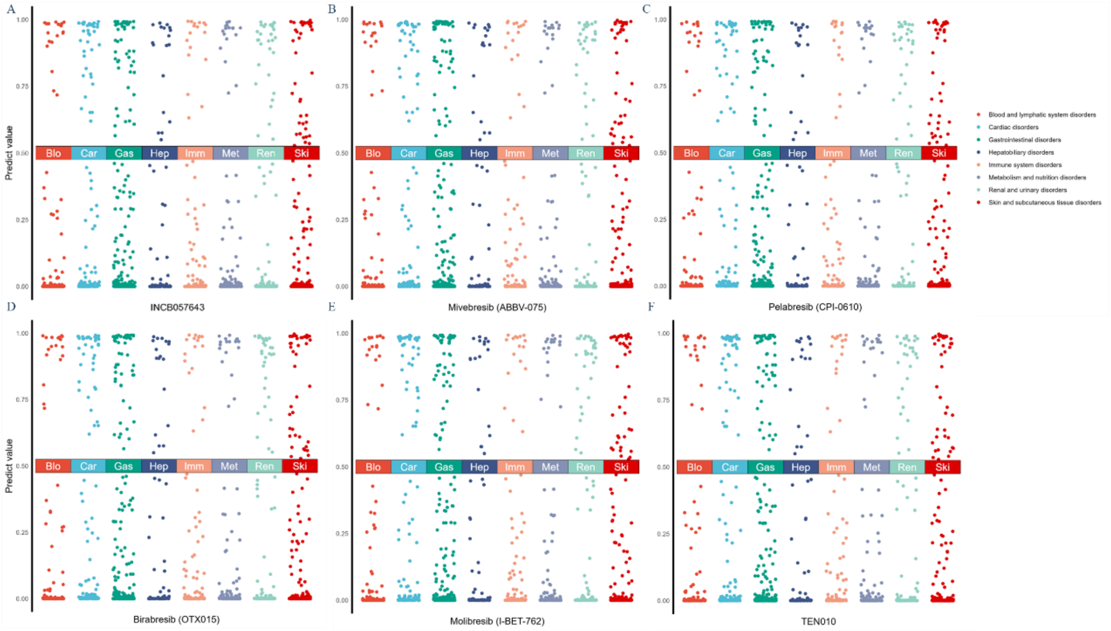

Figure S1. Adverse reaction predictions across different organ systems classifications: (**A**) predictive value for INCB057643 in different system; (**B**) predictive value for Mivebresib in different system; (**C**) predictive value for Pelabresib in different system; (**D**) predictive value for Birabresib in different system; (**E**) predictive value for Molibresib in different system; (**F**) predictive value for TEN010 in different system.

1. Roboz, G.J.; Desai, P.; Lee, S.; Ritchie, E.K.; Winer, E.S.; DeMario, M.; Brennan, B.; Nüesch, E.;

Chesne, E.; Brennan, L. A dose escalation study of RO6870810/TEN-10 in patients with acute myeloid leukemia and myelodysplastic syndrome. *Leukemia & Lymphoma* **2021**, *62*, 1740-1748.
2. Senapati, J.; Fiskus, W.C.; Daver, N.; Wilson, N.R.; Ravandi, F.; Garcia-Manero, G.; Kadia, T.; DiNardo, C.D.; Jabbour, E.; Burger, J. Phase I Results of Bromodomain and Extra-Terminal Inhibitor PLX51107 in Combination with Azacitidine in Patients with Relapsed/Refractory Myeloid Malignancies. *Clinical Cancer Research* **2023**, *29*, 4352-4360.
3. Blum, K.A.; Supko, J.G.; Maris, M.B.; Flinn, I.W.; Goy, A.; Younes, A.; Bobba, S.; Senderowicz, A.M.; Efuni, S.; Rippley, R. A phase I study of pelabresib (CPI-0610), a small-molecule inhibitor of BET proteins, in patients with relapsed or refractory lymphoma. *Cancer research communications* **2022**, *2*, 795-805.
4. Piha-Paul, S.A.; Sachdev, J.C.; Barve, M.; LoRusso, P.; Szmulewitz, R.; Patel, S.P.; Lara Jr, P.N.; Chen, X.; Hu, B.; Freise, K.J. First-in-human study of mivebresib (ABBV-075), an oral pan-inhibitor of bromodomain and extra terminal proteins, in patients with relapsed/refractory solid tumors. *Clinical Cancer Research* **2019**, *25*, 6309-6319.
